# Post‐mortem cardiomegaly descriptor: Call for consistent criteria

**DOI:** 10.1111/1556-4029.70135

**Published:** 2025-07-11

**Authors:** Mark W. Kroll, Dwayne A. Wolf, Klaus Witte, Hugh Calkins, Sebastian N. Kunz, Howard E. Williams

**Affiliations:** ^1^ Biomedical Engineering University of Minnesota Crystal Bay Minnesota USA; ^2^ Deputy Coroner, Lucas County Toledo Ohio USA; ^3^ University of Leeds Leeds UK; ^4^ Johns Hopkins Hospital Baltimore Maryland USA; ^5^ Institute of Forensic Medicine Ulm University Ulm Germany; ^6^ School of Criminal Justice and Criminology Texas State University San Marcos Texas USA

**Keywords:** autopsy, cardiomegaly, diagnosis, forensic pathology, heart disease, sudden cardiac death

## Abstract

Although the post‐mortem descriptor of cardiomegaly is an important component of understanding a sudden death, there is no unified definition. A recent survey reported the usage of heart weight correction models of Molina or Kitzman, for example, or simple step cutoffs such as 350, 400, 450, or 500 g in common use. The goal of the present study was to determine how a diagnosis of cardiomegaly relates to these definitions and heart weight using a database of sudden deaths using 1071 autopsy reports from across the USA in which the heart weight and the presence (*n* = 373) or not (*n* = 698) of cardiomegaly were recorded. We found that medical examiners appear not to use corrections for body weight but instead rely on step weight cutoffs, predominantly of 350, 400, 450, and 500 g. The decedent's age, weight, ethnicity, and toxicology did not tend to influence a diagnosis of cardiomegaly. The term cardiomegaly is being used with increasing frequency with an average increase of 3.6% per year. Consistency in the post‐mortem use of cardiomegaly is lacking.

**Highlights**

- There is no single recognized criterion for the post‐mortem diagnosis of cardiomegaly.
- We found 1071 autopsy reports from 2006 to 2021 with sufficient information for analysis of the diagnosis.
- Medical examiners tend not to use heart weight corrections for body size but appear to rely on step weight cutoffs.
- The cardiomegaly diagnosis is increasing at a rate of 3.6% per year.

## 1 INTRODUCTION

Cardiac enlargement, particularly left ventricular hypertrophy, is recognized during life as a risk for sudden arrhythmic death. Consequently, an increased heart weight is often identified as the cause of death (or contributor to), an unexplained death during post‐mortem examinations [1, 2, 3, 4]. A post‐mortem notice of a heavy heart in adults is a recent phenomenon. In 1901, an autopsy finding of a 530 g heart in a 25‐year‐old male was reported with a lengthy case report, where the heart was described as “le coeur tres hypertrophic” [5]. The term “cardiomegaly” is an even more recent development, having first appeared in paper titles in the 1930s in case reports of infants [6]. The earliest case *series* we found with “cardiomegaly” in the title was Levy's review of normal young adults in 1955 (*n* = 20) [7]. Subsequently, the term “cardiomegaly” has become preferred by pathologists for increased heart weight, and autopsy reports usually include the weight of the heart.

However, there is no definition of “cardiomegaly” in the pathology literature. Moreover, “cardiomegaly” simply means a *large* heart with the most common usage in clinical medicine being in radiology reports where a cardiothoracic ratio of >0.5 is labeled as cardiomegaly [8]. This roughly corresponds to a heart weight being 1.0 SD (standard deviation) above the Zeek normal heart weights (1942) predicted by body length [9]. In contrast to the frequency of the usage of the term “cardiomegaly” by pathologists, it is rarely used by cardiologists since it gives no indication of whether there is hypertrophy (a heavier heart) or dilation (larger dimensions), which also can be linked to specific cardiac diseases. Hence, over the last 50 years, the term “cardiomyopathy” has appeared in 2161 manuscript titles in the two leading clinical cardiology journals—“Journal of the American College of Cardiology” and “Circulation” (American Heart Association)—the term “cardiomegaly” appears in only three titles.

Given the widespread use of the term “cardiomegaly” by U.S. medical examiners, Schoppen surveyed this group regarding their diagnostic criteria for this label [10]. He found considerable variation across the USA, ranging from the routine use of heart weight models of Molina or Kitzman to adjust for body habitus [11, 12, 13] or simpler hard cutoffs varying from 350 to 500 g. Some reported using linear heuristic models such as 5 g/kg of body weight. The Schoppen survey, while exposing great variation in practice, confirms that “cardiomegaly” is used to describe a *heavier* heart and not a dimensionally larger heart as defined by radiologists. The Schoppen paper also included a heart weight model based on autopsy results from 3398 hearts.

The goal of our study was to find how the cardiomegaly descriptor correlates to the various definitions in current usage by forensic pathologists, and also to see if the geographical region, decedent sex, race, and toxicology findings introduce any bias into this labeling. Finally, we sought to evaluate whether there is any long‐term trend in the use of the cardiomegaly descriptor.

## 2 METHODS

The study used the previously described Williams autopsy database of sudden arrest‐related deaths, as it comprises a high prevalence of cardiomegaly diagnoses and was readily available for analysis [14, 15]. Obviously, a database of normal hearts would not have been helpful. We excluded autopsy reports lacking race, heart weight, body height, or body weight, and we limited the analysis to the 16‐year range of 2006–2021, leaving 1071 cases for analysis.

We searched the narrative description of the organs and the summary outline of the findings for the term “cardiomegaly.” Most offices include synoptic reporting in addition to the actual descriptive narrative. Therefore, the term “autopsy report,” includes both the synoptic page as well as the organ description.

We analyzed the post‐mortem cardiomegaly descriptor with logistic regression, using various published models for estimating normal heart weight based on body habitus. There are numerous published studies of heart weight [9, 10, 16, 17, 18, 19, 20, 21, 22]. We tested the most frequently reported four models [11, 12, 13]. The Molina and Kitzman models offer an estimate of normal heart weight based on the subject's body weight (or height, but not both) and sex. Both the Wingren‐Beers and Schoppen models use sex, body height, and weight, along with age.

Hence, our hypothesis was that the ratio of the *actual* heart weight to the model‐*predicted* heart weight would be related to the label of cardiomegaly since this was reported in the Schoppen survey.

All four models were therefore applied to each of the decedents using their characteristics to estimate an expected heart weight for each individual, which was then compared with the actual heart weight to achieve the ratio as described. For example:

$$\begin{array}{c} \text{Kitzman ratio}=\text{actual heart weight}\\ \;÷\text{heart weight predicted}\;\mathrm{by}\;\text{Kitzman model} \end{array}$$

Subsequently, we used this ratio to determine whether the actual weight was higher than expected, by how much, and whether this was related to a cardiomegaly diagnosis. This process was then repeated, including the reported fixed‐weight cutoff steps (such as 350, 400, 450, and 500 g).

We then tested the influence of race, sex, and toxicology findings as potential predictor variables of the cardiomegaly diagnosis. Finally, we tested the possible predictors of autopsy year, and the U.S. geographical region for the Medical Examiner office, where “West” comprised those states west of or including the Rocky Mountains, and “South” comprised the states from Oklahoma to Virginia and below [14]. This was required for model validity due to the reduced popularity of the cardiomegaly diagnosis in the northern states.

## 3 RESULTS

Table 1 shows the demographics of the population split by cardiomegaly diagnosis. Decedents identified as having cardiomegaly had significantly greater height, weight, and BMI. Notable was that males *without cardiomegaly* had similar heart weights to the females *with cardiomegaly* (418 ± 91 vs. 430 ± 78 g; *p* = 0.49), suggesting that medical examiners used different cutoffs for the diagnosis depending on the sex.

**TABLE 1:** Summary data of subject age, body habitus, and heart weight.

	Mean	Min	Median	Max	Cardiomegaly (373)	Controls (698)
Age (years)	38.2 ± 10.8	15	37	87	41.2 ± 10.6	36.6 ± 10.5
Male					512 ± 100	418 ± 91
Female					430 ± 78	338 ± 76
Body height (m)	1.77 ± 0.08	1.40	1.78	2.01	1.79 ± 0.08	1.76 ± 0.08
Body weight (kg)	94.7 ± 24.4	40.8	91.1	239.0	105 ± 25.8	89.1 ± 21.7
BMI (kg/m^2^)	30.1 ± 7.0	15.2	29.3	63.6	32.6 ± 7.6	28.7 ± 6.2
Heart weight (g)	445.8 ± 105.9	220	430	1120	509 ± 100.6	412 ± 92.4

*Note*: All cardiomegaly vs. control differences are significant at *p* < 0.0001 by pooled *T*‐test.

### 3.1 Heart weight models

**FIGURE 1:**
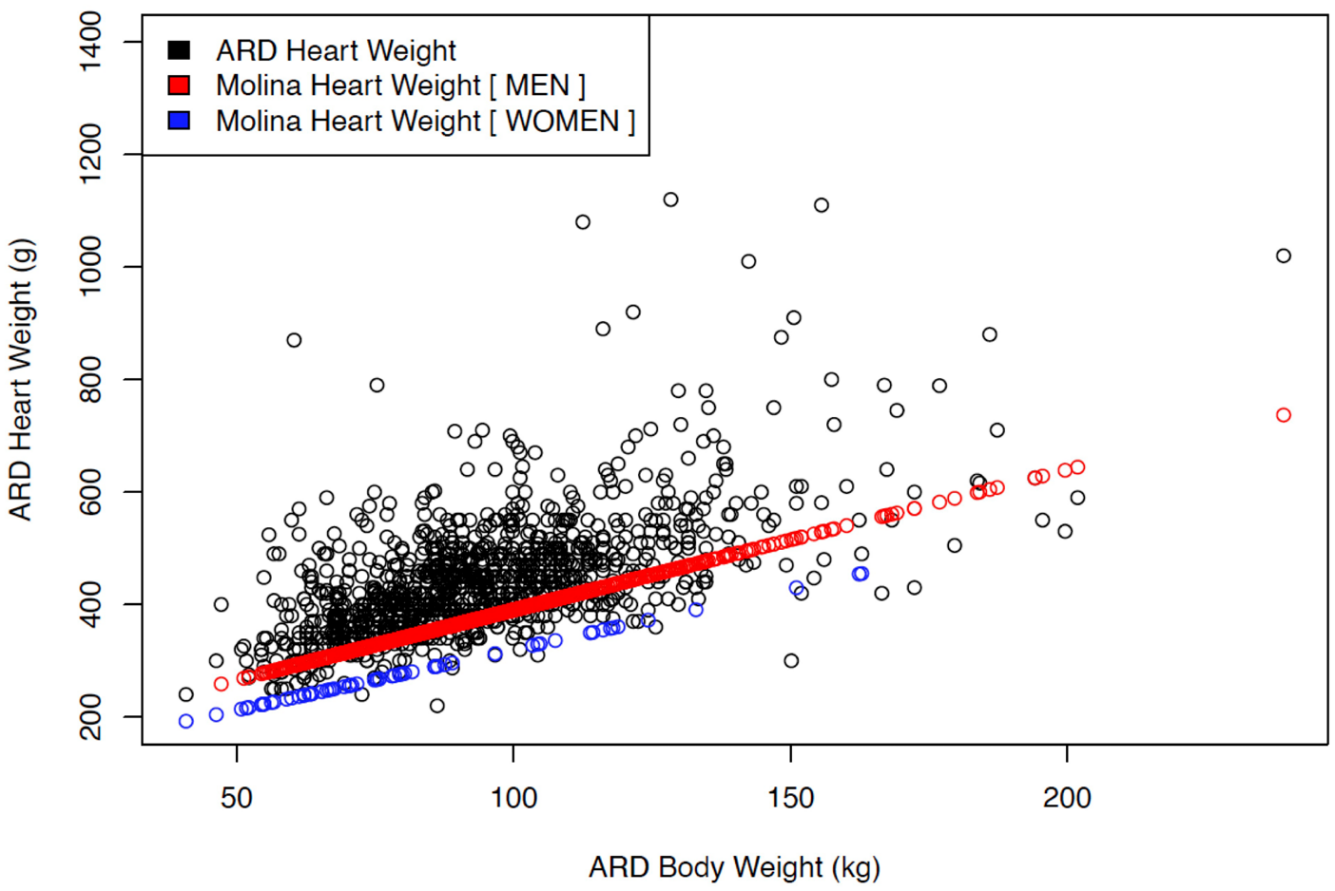
Actual (ARD) heart weights plotted with Molina model for normal heart weight for the decedent's body weight and sex.

Figure 1 shows the *actual* heart weights in the Williams ARD database compared with the predicted weights according to the model of Molina et al., plotted against body weight. Since this model predicts *heart* weight by a simple correlation with *body* weight, there is a linear relationship between *body* weight and expected *heart* weights. Visual inspection shows that this model underpredicts heart weight. The Kitzman model (not shown) had a similar underprediction.

**FIGURE 2:**
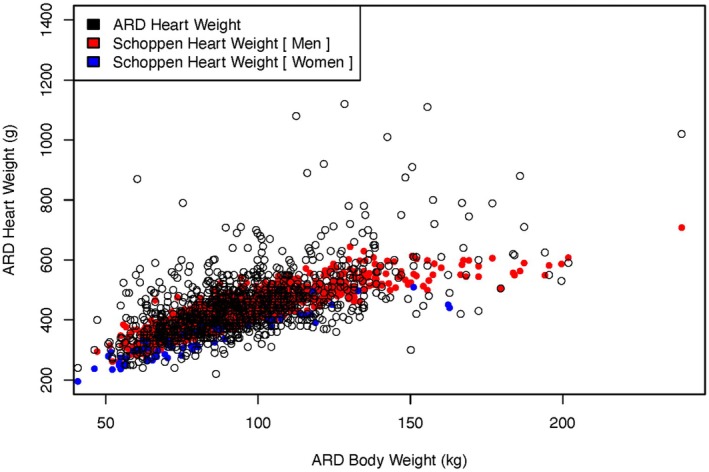
Actual heart weights vs. Schoppen prediction. Note that the predictions are no longer two lines since they incorporate more decedent data than just body weight.

Figure 2 shows the actual weights of the ARD cases compared with the predicted weights according to the model of Schoppen et al. Most males fall within the range proposed by this model, but many have a heart weight above their expected range, suggesting a cardiomegaly diagnosis. The Wingren‐Beer model (not shown) had very similar results.

**FIGURE 3:**
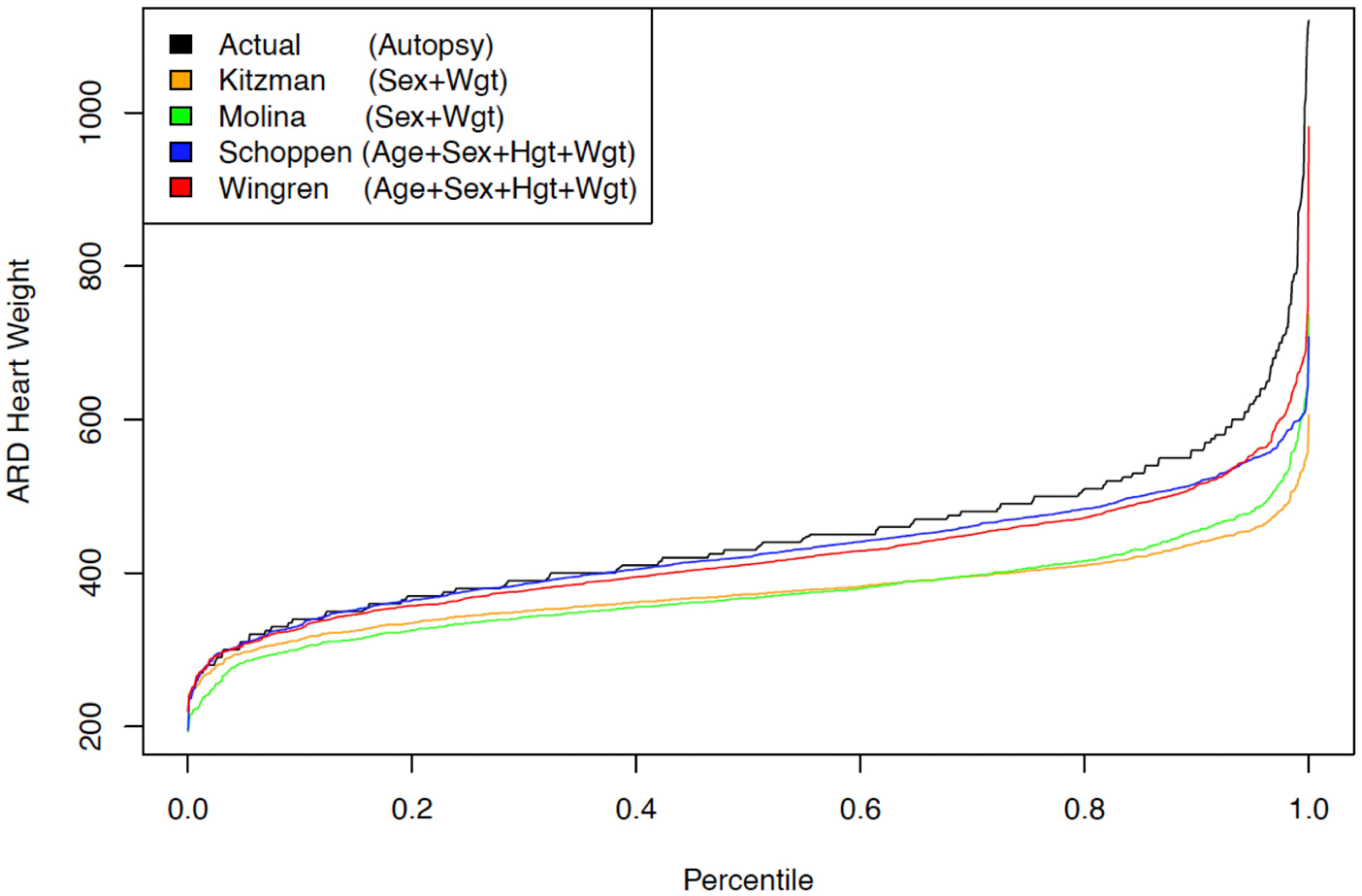
Comparison of Kitzman, Molina, Schoppen, and Wingren models.

To compare the newer (Schoppen and Wingren‐Beer) to the older (Kitzman and Molina) models, we made a cumulative ranked comparison. The expected *normal* estimated heart weight was calculated for each subject, according to each of the four models. As seen in Figure 3, the Schoppen and Wingren models accurately track the ARD heart weights up to about the 60th percentile. Given the frequency of cardiomegaly in the Williams ARD database, the relationship between expected and actual becomes less reliable as heart weights increase.

In the initial logistic regression analysis, none of the heart weight model *ratios* using the predicted weights were statistically linked with a cardiomegaly diagnosis. On the other hand, the *actual* heart weight was a significant predictor, suggesting that the actual heart weight was favored (vs. cutoffs based on the body size) in making a diagnosis of cardiomegaly.

### 3.2 Step weight cutoffs

**FIGURE 4:**
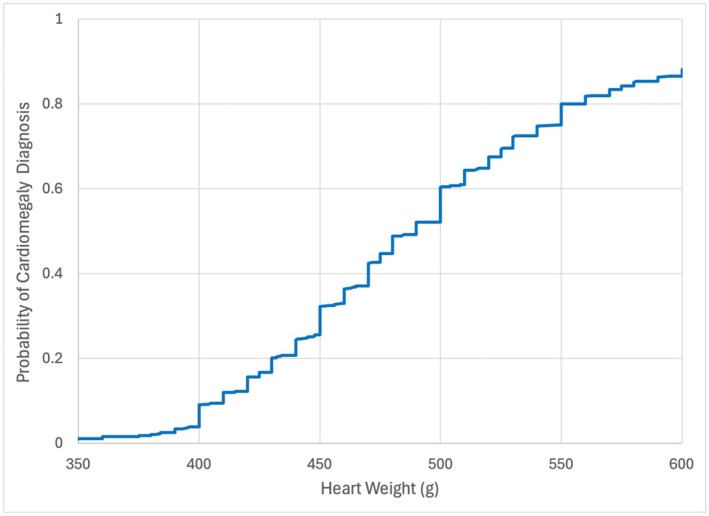
Cardiomegaly labeling as a function of heart weight.

Figure 4 shows visual evidence of the “step” weight cutoffs, where we plotted the probability of the cardiomegaly descriptor as a function of heart weight. The large steps at 400, 450, and 500 g are visibly obvious and intuitively suggest the popularity of hard step cutoffs. The heart weight is almost always recorded to the nearest 10 g, and thus there are small statistically insignificant artifactual steps seen at every 10 g increment. They should not be confused with diagnostic cutoffs. To test our hypothesis that step cutoffs are used, we performed a logistic regression, adding multiple step cutoff possibilities to the original ratio:

$$\begin{array}{c} \text{Cardiomegaly descriptor}\\ =f\;\left(\text{Ratio},350\;\text{step},400\;\text{step},450\;\text{step},500\;\text{step},550\;\text{step},600\;\text{step}\right) \end{array}$$

The step functions for cutoffs of 350, 400, 450, and 500 g were consistently related to the diagnosis of cardiomegaly as shown in Table 2. On the other hand, the Schoppen ratio and the 550 g and 600 g steps were not. We then repeated this analysis for the Wingren‐Beer, Kitzman, and Molina ratios and had similar results, suggesting that medical examiners tend to use absolute step cutoffs and apparently do not typically use the heart weight prediction models to establish a diagnosis of cardiomegaly.

**TABLE 2:** Model using decedent, region, and year predictors.

	Estimate	SE	*p*
(Intercept)	−4.98	0.69	<0.0001
500 g step	0.75	0.21	0.0004
450 g step	0.77	0.20	<0.0001
400 g step	1.40	0.25	<0.0001
350 g step	2.12	0.63	<0.0001
Region	0.18	0.09	0.038
Year	0.036	0.017	0.035

### 3.3 Other prediction variables

Decedent age, sex, and race were not statistically significant predictors of a diagnosis of “cardiomegaly” in the Williams database. Toxicology findings of methamphetamine, cocaine, and alcohol also did not influence the use of the “cardiomegaly” label. The reduced popularity of the cardiomegaly term, in the northern states, was reflected and included in the model. Moreover, the usage of the cardiomegaly diagnosis was increasing by 3.6% per year of death (*p* = 0.035). Over the 16‐year timeframe of the study, the mean heart weight (447 ± 105 g) did not increase. Due to the non‐Gaussian distribution from the many large hearts, a useful comparison value is the median weight of 430 g [IQR: 380–497].

### 3.4 Bivariate model

**FIGURE 5:**
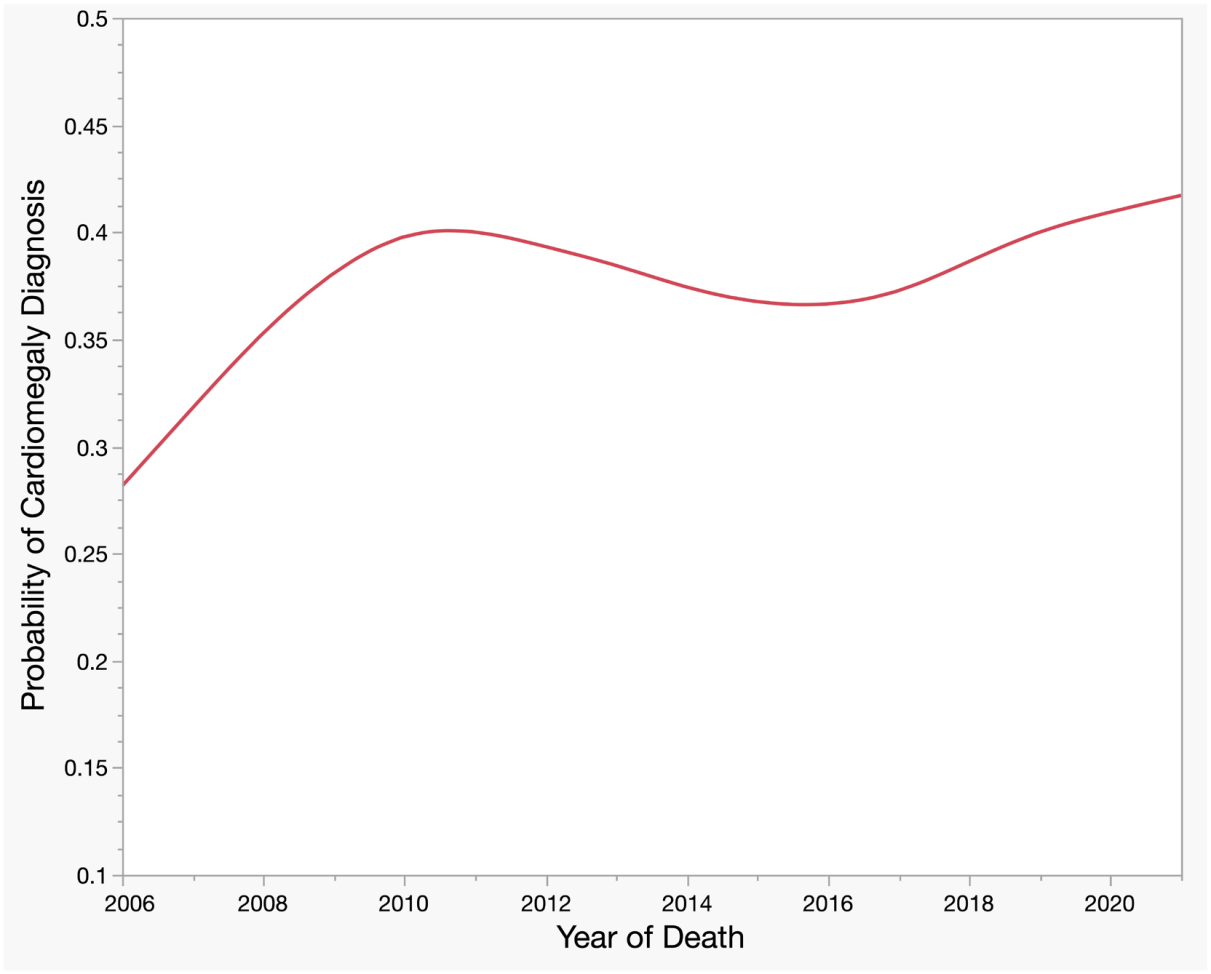
Cardiomegaly diagnosis as a function of year of death.

Due to the unexpected emergence of the year of death as a significant predictor in the multivariate analysis, we elected to do a bivariate analysis using only the year of death and actual heart weight. The year of death was again a significant predictor—of the cardiomegaly descriptor—at 3.6% per year (*p* = 0.02), and the heart weight increased the descriptor at 1.2% per gram (*p* < 0.0001). See Figure 5 for a plot of the cardiomegaly diagnosis versus year of death.

## 4 DISCUSSION

This study finds, for the first time, that Medical Examiners in the USA do not tend to incorporate body weight or height into a diagnosis of “cardiomegaly” (increased heart weight) in an autopsy report, preferring instead to compare the weight of the decedent heart with standard weight cutoffs.

Our logistic regression modeling of the cardiomegaly diagnosis suggests that—contrary to previous surveys—heart weight prediction models are not in common use [10]. This could be the result of conflicting information in the literature where hard cutoffs are reportedly preferred, even by those who have developed such a model [10, 11]. We did find that hard cutoffs for a heavy heart of 350, 400, 450, and 500 g were commonly used. This is consistent with the Schoppen survey finding that only those same four cutoffs had been used for male hearts. The fact that other decedent characteristics (age, ethnicity, and toxicology) had no influence reinforces our finding that medical examiners focus primarily on the heart weight and sex when determining a cardiomegaly diagnosis.

For an upper limit of normality, Schoppen et al., proposed the 95th percentile. This is equivalent to a deviation of 28% (1.65 SD) from the absolute calculated expected heart weight.

An upper limit cutoff of 1.0 SD above the predicted heart weight from a model such as that of Wingren‐Beer or Schoppen would give a consistent post‐mortem descriptor of cardiomegaly [10, 22]. Both the Schoppen and Wingren models have online calculators: https://labs.feinberg.northwestern.edu/webster/heart_weight/ lundforensicmedicine.com.

This cutoff is easily calculated by taking 117% of the modeled normal heart weight for either the Wingren‐Beer or the Schoppen model, where the 1.17 ratio matches a 1.0 SD departure from the expected heart weight. Moreover, 1.0 SD would be consistent with the radiology definition of cardiomegaly [8, 23].

An unexpected finding was that the use of the cardiomegaly diagnosis is increasing over time. The current analysis does not allow for an exploration of the underlying causes of this change in approach, but it is possible that an increasing awareness of the arrhythmic risks of cardiac hypertrophy could be contributing.

Our database revealed that the term “cardiomegaly” remains in common usage, and although it is formally translated as “big heart” its persistence in autopsy reports as a descriptor for a heavy heart should be considered. In the clinical cardiology field, the term cardiomegaly is not in common usage but would hint at a form of cardiomyopathy, which comes with a considerably increased risk of sudden death. On the other hand, a heavy heart is typically described using the term “hypertrophy” in the clinical setting. This descriptor comes with a typical appearance very different from a dilated but not hypertrophied heart and with very different potential etiologies, with a lower but not normal arrhythmic risk. Typically, forensic pathologists first describe a heart as heavy (cardiomegaly), and then describe why (left ventricular hypertrophy, etc).

Radiologists diagnose cardiomegaly on the chest radiograph when the cardiothoracic ratio exceeds 0.5. This had a weak correlation to left ventricular ejection fraction (or other cardiac pathology) or magnetic‐resonance calculated cardiac mass in 309 subjects [23]. A comparison of the cardiothoracic ratio to radionuclide ventriculography and echocardiography also found minimal correlations and an inability to predict ventricular function [24].

### 4.1 Limitations

We used a database of arrest‐related sudden death autopsy reports, as that was readily accessible to us and had a significant prevalence of cardiomegaly findings. We predict that using autopsy reports from other causes of sudden death (motor vehicle accidents, for example) would have different heart weights. We do not believe that this detracted from our statistical analysis, as the inclusion of many large hearts provided greater statistical leverage for our conclusions.

We made no allowance for the different ways heart weights are assessed. While it is considered best practice to remove the aortic arch and luminal blood before weighing the heart, this does not happen in all medical examiner's facilities. This is unlikely to have had an effect on our analysis since our goal was to see what *weight cutoffs* forensic pathologists used based on the weight that they determined by whatever method they had for preparing the heart before placing it on the scale.

There can be variation in the practice of forensic pathologists in using this term among their own cases. For example, a decedent who dies from a traumatic brain injury may have a 650 g heart weight listed in the internal examination without a callout as cardiomegaly, as it was not relevant to the death, while another individual with a 650 g heart who dies in a situation in which the heart weight is considered pathologic and causative or contributory to the death may have it called out as cardiomegaly in the final diagnoses.

We could have added terms such as “hypertensive cardiovascular disease” as somewhat synonymous with cardiomegaly, but chose not to, as the point of our analysis was to look for consistency in terminology as well as diagnosis. A diagnosis of cardiomegaly is not necessarily a reliable indicator of whether the autopsying pathologist considered the heart to be increased in weight.

## 5 CONCLUSIONS

In the labeling of cardiomegaly, American medical examiners tend not to use corrections for body habitus or other variables, and our data suggest that they tend to rely on hard step‐cutoffs predominantly of 350, 400, 450, and 500 g. For the same heart weights, the diagnosis of “cardiomegaly” was increasing with an average increase of 3.6% per year.

## CONFLICT OF INTEREST STATEMENT

KW, SNK, & HC are members of the AXON Scientific and Medical Advisory Board (SMAB). MWK was previously a member of the AXON Corporate Board and SMAB. MWK and HC have been expert witnesses in litigation.

## Data Availability

Data available upon reasonable request.
